# Perspectives on Individual Animal Identification from Biology and Computer Vision

**DOI:** 10.1093/icb/icab107

**Published:** 2021-05-29

**Authors:** Maxime Vidal, Nathan Wolf, Beth Rosenberg, Bradley P Harris, Alexander Mathis

**Affiliations:** 1 School of Life Sciences, Brain Mind Institute, Swiss Federal Institute of Technology (EPFL), Chemin des Mines 9, 1202 Geneva, Switzerland; 2 Center for Neuroprosthetics, Center for Intelligent Systems, Swiss Federal Institute of Technology (EPFL), Chemin des Mines 9, 1202 Geneva, Switzerland; 3 Fisheries, Aquatic Science, and Technology Laboratory, Alaska Pacific University, 4101 University Drive, Anchorage, Alaska 99508, USA

## Abstract

Identifying individual animals is crucial for many biological investigations. In response to some of the limitations of current identification methods, new automated computer vision approaches have emerged with strong performance. Here, we review current advances of computer vision identification techniques to provide both computer scientists and biologists with an overview of the available tools and discuss their applications. We conclude by offering recommendations for starting an animal identification project, illustrate current limitations, and propose how they might be addressed in the future.

## Introduction

The identification^1^ of specific individuals is central to addressing many questions in biology: does a sea turtle return to its natal beach to lay eggs? How does a social hierarchy form through individual interactions? What is the relationship between individual resource use and physical development? Indeed, the need for identification in biological investigations has resulted in the development and application of a variety of identification methods, ranging from physical tags (Rácz et al. 2021) to genetic methods (Palsbøll 1999; John 2012), GPS tracking (Baudouin et al. 2015), and radio-frequency identification (Bonter and Bridge 2011; Weissbrod et al. 2013). While each of these methods is capable of providing reliable re-identification, each is also subject to limitations, such as invasive implantation or deployment procedures, high costs, or demanding logistical requirements. Image-based identification techniques using photos, camera-traps, or videos offer (potentially) low-cost and non-invasive alternatives. However, identification success rates of image-based machine analyses have traditionally been lower than many of the aforementioned alternatives. Nonetheless, experts can perform this task very well (e.g., Jouke et al. 2020), further motivating computer vision approaches.

Using computer vision to identify animals dates back to the early 1990s and has developed quickly since (see Schneider et al. (2019) for an excellent historical account). The advancement of new machine learning tools, especially deep learning (LeCun et al. 2015; Norouzzadeh et al. 2018; Schneider et al. 2019; Mathis et al. 2020; Xiongwei et al. 2020), offers powerful methods for improving the accuracy of image-based identification analyses. In this review, we introduce relevant background for animal identification with deep learning based on visual data, review recent developments, identify remaining challenges, and discuss the consequences for biology, including ecology, ethology, neuroscience, and conservation modeling. We aimed to create a review that can act as a reference for researchers who are new to animal identification and can also help current practitioners interested in applying novel methods to their identification work.

## Biological context for identification

Conspecific identification is crucial for most animals to avoid conflict, establish hierarchy, and mate (e.g., Hagey and Macdonald 2003; Martin et al. 2008; Levréro et al. 2009). For some species, it is understood how they identify other individuals—for instance, penguin chicks make use of the distinct vocal signature based on frequency modulation to recognize their parents within enormous colonies (Jouventin et al. 1999). However, for many species, the mechanisms of conspecific identification are poorly understood. What is certain is that animals use multiple modalities to identify each other, from audition, to vision and chemosensation (Hagey and Macdonald 2003; Martin et al. 2008; Levréro et al. 2009). Much like animals use different sensors, techniques using different modalities have been proposed for identification. From the technical point of view, the selection of characteristics for animal identification (termed *biometrics*) is primarily based on universality, uniqueness, permanence, measurability, feasibility, and reliability (Jain et al. 2007). More specifically, reliable biometrics should display little intra-class variation and strong inter-class variation. Fingerprints, iris scans, and DNA analysis are some of the well-established biometric methods used to identify humans (Palsbøll 1999; Jain et al. 2007; John 2012). However, other physical, chemical, or behavioral features such as gait patterns may be used to identify animals based on the taxonomic focus and study design (Jain et al. 2007; Kühl and Burghardt 2013). For the purposes of this review, we will focus on visual biometrics and what is currently possible.

## Visual biometrics: framing the problem

What are the key considerations for selecting potential “biometric” markers in images? We believe they are: (1) a strong differentiation among individuals based on their visible traits and (2) the reliable presence of these permanent features by the species of interest within the study area. Furthermore, one should also consider whether they will be applied to a closed or open set (Jonathon Phillips and Grother 2011). Consider a fully labeled dataset of unique individuals. In closed set identification, the problem consists of images of multiple, otherwise known, individuals, who shall be “found again” in (novel) images. In the more general and challenging case of open set identification, the (test) dataset may contain previously unseen individuals, thus permitting the formation of new identities. Depending on the application, both of these cases are important in biology and may require the selection of different computational methods. Open-set identification in general is an unsolved problem, as long-tail distributions (of individuals) stymies fine-grained discrimination.

## Animal identification: the computer vision perspective

**Fig. 1:**
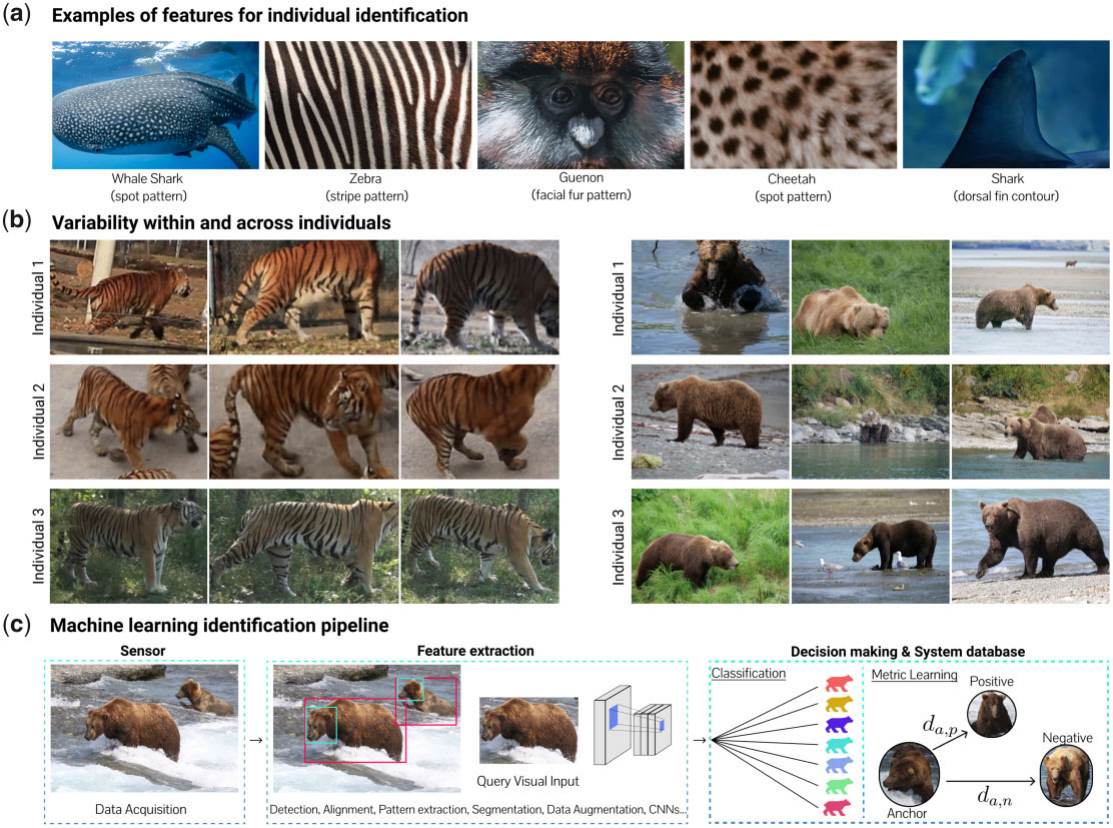
(**a**) Animal biometrics examples featuring unique distinguishable phenotypic traits (adapted with permission from unsplash.com). (**b**) Three pictures each of three example tigers from the Amur Tiger reID Dataset (Shuyuan et al. 2019) and three pictures each of three example bears from the McNeil River State Game Sanctuary (photo credit Alaska Department of Fish and Game). The tiger stripes are robust visual biometrics. The bear images highlight the variations across seasons (fur and weight changes). Postures and contexts vary more or less depending on the species and dataset and further complicate identification. (**c**) Machine learning identification pipeline from raw data acquisition through feature extraction to identity retrieval.

Some animals have specific visual traits, such as characteristic fur patterns, a property that greatly simplifies visual identification, while other species lack a salient, distinctive appearance (Fig. 1a and b). Apart from visual appearance, additional challenges complicate animal identification, such as changes to the body over time, environmental changes and migration, deformable bodies, variability in illumination and view, as well as obstruction (Fig. 1b).

Computational pipelines for animal identification consist of a sensor and modules for feature extraction, decision-making, and a system database (Fig. 1c; Jain et al. 2007). Sensors, typically cameras, capture images of individuals which are transformed into salient, discriminative features by the feature extraction module. In computer vision, a feature is a distinctive attribute of the content of an image (at a particular location). Features might be, for example, edges, textures, or more abstract attributes. The decision-making module uses the computed features to identify the most similar known identities from the system database module, and in some cases, assign the individual to a new identity.

For many other tasks, such as animal localization, species classification and pose estimation, computer vision pipelines follow similar principles (see Box 1 for more details on those systems). As we will illustrate below, many of these tasks also play an important role in identification pipelines; for instance animal localization and alignment is a common component (see Fig. 1c).

In order to quantify identification performance, let us define the relevant evaluation metrics. These include top-*N* accuracy, that is, the frequency of the true identity being within the *N* most confident predictions, and the mean average precision (mAP) defined in Box 2. A perfect system would demonstrate a top-1 score and mAP of 100%. However, animal identification through computer vision is a challenging problem, and as we will discuss, algorithms typically fall short of this ideal performance. Research often focuses on one species (and dataset), which is typically encouraged by the available data. Overall, few benchmarks have been established, and adding to the varying difficulty and variability of the different datasets, different evaluation methods and train-test splits are used, making the comparison between the different methods arduous and the performance dependent on the architecture–dataset pair. Thus, one must proceed with extreme caution when comparing publications to each other, if working with a different species, or a different dataset of the same species. We hope that future work will focus on standardizing evaluation protocols, and sharing data and code, so that results can be straightforwardly compared.

As reviewed by Schneider et al. (2019), the use of computer vision for animal identification dates back to the early 1990s. This recent review also contains a comprehensive table summarizing the major milestones and publications. In the meantime, the field has further accelerated, and we provide a table with salient animal identification datasets since its publication (Table 1).

**Table 1:** Recent animal identification publications and relevant data

Method	Species	Target	Identities	Train Images	Test Images	Results
Chen et al. (2020)	Panda	Face	218	5845	402	Top-1: 96.27^a^
Shuyuan et al. (2019)	Tiger (ATRW)	Body	92	1887	1762	Top-1: 88.9, Top-5: 96.6, mAP: 71.0^b^
Liu et al. (2019)	Tiger (ATRW)	Body	92	1887	1762	Top-1: 95.6, Top-5: 97.4, mAP: 88.9^b^
Moskvyak et al. (2019)	Manta Ray	Underside	120	1380	350	Top-1: 62.05 ± 3.24, Top-5: 93.65 ± 1.83
Moskvyak et al. (2019)	Humpback Whale	Fluke	633	2358	550	Top-1: 62.78 ± 1.6, Top-5: 93.46 ± 0.63
Bouma et al. (2018)	Common Dolphin	Fin	180	∼2800	∼700	Top-1:90.5±2, Top-5: 93.6±1
Nepovinnykh et al. (2020)	Saimaa Ringed Seal	Pelage	46	3000	2000	Top-1: 67.8, Top-5: 88.6
Schofield et al. (2019)	Chimpanzee	Face	23	3,249,739	1,018,494	Frame-acc : 79.12%, Track-acc: 92.47%
Clapham et al. (2020)	Brown Bear	Face	132	3740	934	Acc: 83.9%

This table extends the excellent list in Schneider et al. (2019) by subsequent publications.

^a^ Closed set.

^b^ Single camera wild.

In computer vision, features are the components of an image which are considered significant. In the context of animal identification pipelines (and computer vision more broadly), two classes of features can be distinguished. Handcrafted features are a class of image properties that are manually selected (a process known as “feature engineering”) and then used directly for matching or computationally utilized to train classifiers. This stands in contrast to deep features which are automatically determined using learning algorithms to train hierarchical processing architectures based on data (LeCun et al. 2015; Mathis et al. 2020; Xiongwei et al. 2020). In the following sections, we will structure the review of relevant papers depending on the use of handcrafted and deep features. We also provide a glossary of relevant machine learning terms in Box 2.

## Handcrafted features

The use of handcrafted features is a powerful, classical computer vision method, which has been applied to many different species that display unique, salient visual patterns, such as zebras' stripes (Lahiri et al. 2011), cheetahs’ spots (Kelly 2001), and guenons’ face marks (Allen and Higham 2015; Fig. 1a). Hiby et al. (2009) exploited the properties of tiger stripes to calculate similarity scores between individuals through a surface model of tigers’ skins. The authors report high model performance estimates (a top-1 score of 95% and a top-5 score of 100% on 298 individuals). It is notable that this technique performed well despite differences in camera angle of up to 66 degrees and image collection dates of 7 years, both of which serve to illustrate the strength of this approach. In addition to the feature descriptors used to distinguish individuals by fur patterns, these models may also utilize edge detectors, thereby allowing individual identification of marine species by fin shape. Indeed, Hughes and Burghardt (2017) employed edge detection to examine great white shark fins by encoding fin contours with boundary descriptors. The authors achieved a top-1 score of 82%, a top-10 score of 91%, and a mAP of 0.84 on 2456 images of 85 individuals (Hughes and Burghardt 2017). Similarly, Weideman et al. (2017) used an integral curvature representation of cetacean flukes and fins to achieve a top-1 score of 95% using 10,713 images of 401 bottlenose dolphins and a top-1 score of 80% using 7173 images of 3572 humpback whales. Furthermore, work on great apes has shown that both global features (i.e., those derived from the whole image) and local features (i.e., those derived from small image patches) can be combined to increase model performance (Alexander 2012; Loos and Ernst 2013). Local features were also used in Crouse et al. (2017), who achieved top-1 scores of 93.3%±3.23% on a dataset of 462 images of 80 individual red-bellied lemurs. Prior to matching, the images were aligned with the help of manual eye markings. Extracting contours using classic algorithms from images can be challenging—recently, Weideman et al. used deep learning to more robustly extract contours, which improved identification of elephants and humpback whales (Hendrik et al. 2020).

Common handcrafted features are designed to extract salient, invariant features from images can also be utilized; a classical example is the scale-invariant feature transform (Lowe 2004). Building upon this, instead of focusing on a single species, Crall et al. (2013) developed HotSpotter, an algorithm able to use stripes, spots, and other patterns for the identification of multiple species.

As these studies highlight, for species with highly discernible physical traits, handcrafted features have shown to be accurate but often lack robustness. Deep learning has strongly improved the capabilities for animal identification, especially for species without clear visual traits. However, as we will discuss, hybrid systems have emerged recently that combine handcrafted features and deep learning.

## Deep features

In the last decade, deep learning, a subset of machine learning in which decision-making is performed using learned features generated algorithmically (e.g., empirical risk minimization with labeled examples; Box 2) has emerged as a powerful tool to analyze, extract, and recognize information. This emergence in large part is due to increases in computing power, the availability of large-scale datasets, open-source and well-maintained deep learning packages, and advances in optimization and architecture design (LeCun et al. 2015; Schneider et al. 2019; Xiongwei et al. 2020). Large datasets are ideal for deep learning, but data augmentation, transfer learning, and other approaches reduce the thirst for data (LeCun et al. 2015; Schneider et al. 2019; Mathis et al. 2020; Xiongwei et al. 2020). Data augmentation is a way to artificially increase dataset size by applying image transformations such as cropping, translating, rotating, as well as incorporating synthetic images (LeCun et al. 2015; Mathis et al. 2020; Beery et al. 2020). Since identification algorithms should be robust to those changes, augmentation often improves performance.

Deep learning models can learn multiple increasingly complex representations within their progressively deeper layers and can achieve high discriminative power. Furthermore, as deep features do not need to be specifically engineered and are learned correspondingly for each unique dataset, deep learning provides a potential solution for many of the challenges typically faced in individual animal identification. Such challenges include species with few natural markings, inconsistencies in markings (caused by changes in pelage, scars, etc.), low-resolution sensor data, odd poses, and occlusions. Two methods have been widely used for animal identification with deep learning: classification and metric learning.

## Classification models

In the classification setting, a class (identity) from a set number of classes is probabilistically assigned to the input image. This assignment decision comes after the extraction of features usually done by convolutional neural networks (ConvNets), a class of deep learning algorithms typically applied to image analyses. Note that the input to ConvNets can be the raw images, but also the processed handcrafted features. In one of the first appearances of ConvNets for individual animal classification, Freytag et al. (2016) improved upon work by Loos and Ernst (2013) by increasing the accuracy with which individual chimpanzees could be identified from two datasets of cropped face images (C-Zoo and C-Tai) from 82.88±1.52% and 64.35±1.39% to 91.99±1.32% and 75.66±0.86%. Freytag et al. (2016) used linear support vector machines (SVMs) to differentiate features extracted by AlexNet, a popular ConvNet (Krizhevsky et al. 2012). They also tackled additional tasks including sex prediction and age estimation. Subsequent work by Brust et al. (2017) also used AlexNet features on cropped faces of gorillas, and SVMs for classification. They reported a top-5 score of 80.3% with 147 individuals and 2500 images. A similar approach was developed for elephants by Körschens et al. (2018). The authors used the YOLO object detection network (Redmon et al. 2016) to automatically predict bounding boxes around elephants’ heads (see Box 1). Features were then extracted with a ResNet50 (He et al. 2016) ConvNet, and projected to a lower-dimensional space by principal component analysis, followed by SVM classification. On a highly unbalanced dataset (i.e., highly uneven numbers of images per individual) consisting of 2078 images of 276 individuals, Körschens et al. (2018) achieved a top-1 score of 56% and a top-10 score of 80%. This increased to 74 and 88% for top-1 and top-10, respectively, when two images of the individual in question were used in the query. In practice, it is often possible to capture multiple images of an individual, for instance with camera traps, hence multi-image queries should be used when available.

Other examples of ConvNets for classification include work by Deb et al. (2018), who explored both open- and closed-set identification for 3000 face images of 129 lemurs, 1450 images of 49 golden monkeys, and 5559 images of 90 chimpanzees. The authors used manually annotated landmarks to align the faces, and introduced the PrimNet model architecture, which outperformed previous methods (e.g., Schroff et al. 2015 and Crouse et al. 2017 that used handcrafted features). Using this method, Deb et al. (2018) achieved 93.76±0.90%, 90.36±0.92% and 75.82±1.25% accuracy for lemurs, golden monkeys, and chimpanzees, respectively, for the closed-set. Finally, Chen et al. (2020) demonstrated a face classification method for captive pandas. After detecting the faces with Faster-RCNN (Ren et al. 2017), they used a modified ResNet50 (He et al. 2016) for face segmentation (binary mask output), alignment (outputs are the affine transformation parameters), and classification. They report a top-1 score of 96.27% on a closed set containing 6441 images from 218 individuals. Chen et al. (2020) also used the Grad-CAM method (Selvaraju et al. 2019), which propagates the gradient information from the last convolutional layers back to the image to visualize the neural networks’ activations, to determine that the areas around the pandas’ eyes and noses had the strongest impact on the identification process.

While the examples presented thus far have employed still images, videos have also been used for deep learning-based animal identification. Unlike single images, videos have the advantage that neighboring video frames often show the same individuals with slight variations in pose, view, and obstruction. While collecting data, one can gather more images in the same time-frame (at the cost of higher storage). For videos, Schofield et al. (2019) introduced a complete pipeline for the identification of chimpanzees, including face detection (with a single shot detector; Liu et al. 2016), face tracking (Kanade–Lucas–Tomasi tracker), sex and identity recognition (classification problem through modified VGG-M architectures; Chatfield et al. 2014), and social network analysis. The video format of the data allowed the authors to maximize the number of images per individual, resulting in a dataset of 20,000 face tracks of 23 individuals. These amounts to 10,000,000 face detections, resulting in a frame-level accuracy of 79.12% and a track-level accuracy of 92.47%. The authors also use a confusion matrix to inspect which individuals were identified incorrectly and reasons for this error. Perhaps unsurprisingly, juveniles and (genetically) related individuals were the most difficult to separate. In follow-up work, Bain et al. (2019) were able to predict identities of all individuals in a frame instead of predicting from face tracks. The authors showed that it is possible to use the activations of the last layer of a counting ConvNet (i.e., whose goal is to count the number of individuals in a frame) to find the spatial regions occupied by the chimpanzees. After cropping, the regions were fed into a fine-grained classification ConvNet. This resulted in similar identification precision compared to using only the face or the body, but a higher recall.

In laboratory settings and for videos, tracking is a common approach to identify individual animals and is the process of locating moving objects over time using a camera (Weissbrod et al. 2013; Dell et al. 2014). Recent tracking system, such as idtracker.ai (Romero-Ferrero et al. 2019), TRex (Walter and Couzin 2021), and DeepLabCut (Lauer et al. 2021) have demonstrated the ability to track individuals in groups of lab animals (fish, mice, etc.) by combining tracking with a ID-classifying ConvNet.

## (Deep) metric learning

Most recent studies on identification have focused on deep metric learning, a technique that seeks to automatically learn how to measure similarity and distance between deep features. Deep metric learning approaches commonly employ methods such as siamese networks or triplet loss (Box 2). Schneider et al. (2020) found that triplet loss always outperformed the siamese approach in a recent study considering a diverse group of five different species (humans, chimpanzees, humpback whales, fruit flies, and Siberian tigers); thereby they also tested many different ConvNets, and metric learning always gave better results. Importantly, metric learning frameworks naturally are able to handle open datasets, thereby allowing for both re-identification of a known individual and the discovery of new individuals.

Competitions often spur progress in computer vision (Mathis et al. 2020; Xiongwei et al. 2020). In 2019, the first large-scale benchmark for animal identification was released (example images in Fig. 1b). It poses two identification challenges on the ATRW tiger dataset: plain, where images of tigers are cropped and normalized with manually curated bounding boxes and poses, and wild, where the tigers first have to be localized and then identified (Shuyuan et al. 2019).

The authors of the benchmark also evaluated various baseline methods and showed that metric learning was better than classification. Their strongest method was a pose part-based model, which based on the pose estimation subnetwork processes the tiger image in seven parts to get different feature representations and then used triplet loss for the global and local representations. On the single-camera, wild setting, the authors reported a mAP of 71.0, a top-1 score of 88.9%, and a top-5 score of 96.6% from 92 identities in 8076 videos (Shuyuan et al. 2019). Fourteen teams submitted methods and the best contribution for the competition, developed a novel triple-stream framework (Liu et al. 2019). The framework has a full image stream together with two local streams (one for the trunk and one for the limbs, which were localized based on the pose skeleton) as an additional task. However, they only required the part streams during training, which, given that pose estimation can be noisy, is particularly fitting for tiger identification in the wild. Liu et al. (2019) also increased the spatial resolution of the ResNet backbone (He et al. 2016). Higher spatial resolution is also commonly used for other fine-grained tasks such as human re-identification, segmentation (Chen et al. 2018), and pose estimation (Cheng et al. 2020; Mathis et al. 2020). With these modification, the authors achieved a top-1 score of 95.6% for single-camera wild-ID and a score of 91.4% across cameras.

Metric learning has also been used for mantas with semi-hard triplet mining (Moskvyak et al. 2019). Human-assembled photos of mantas’ undersides (where they have unique spots) were fed as input to a ConvNet. Once the embeddings were created, Moskvyak et al. (2019) used the *k*-nearest neighbors (k-NN) algorithm for identification. The authors achieved a top-1 score of 62.05±3.24% and top-5 of 93.65±1.83% using a dataset of 1730 images of 120 mantas. Replicating the method for humpback whales’ flukes, the authors report a top-1 score of 62.78±1.6% and a top-5 score of 93.46±0.63% using 2908 images of 633 individual whales. Similarly, Bouma et al. (2018) used batch hard triplet loss to achieve top-1 and top-5 scores of 90.5±2% and 93.6±1%, respectively, on 3544 images of 185 common dolphins. When using an additional 1200 images as distractors, the authors reported a drop of 12% in the top-1 score and 2.8% in the top-5 score. The authors also explore the impact of increasing the number of individuals and the number of images per individual, both leading to score increases. Nepovinnykh et al. (2020) applied metric learning to re-identify Saimaa ringed seals. After segmentation with DeepLab (Chen et al. 2018) and subsequent cropping, the authors extracted pelage pattern features with a Sato tubeness filter used as input to their network. Indeed, Kshitij and Sai (2020) also showed that—for some species—priming ConvNets with handcrafted features produced better results than using the raw images. Instead of using k-NNs, Nepovinnykh et al. (2020) adopt topologically aware heatmaps to identify individual seals—both the query image and the database images are split into patches whose similarity is computed, and among the most similar, topological similarity is checked through angle difference ranking. For 2000 images of 46 seals, the authors achieved a top-1 score of 67.8% and a top-5 score of 88.6%. Overall, these recent papers highlight that recent work has combined handcrafted and deep learning approaches to boost the performance.

## Applications of animal identification in field and laboratory settings^3^

Here, we discuss the use of computer vision techniques for animal identification from a biological perspective and offer insights on how these techniques can be used to address broad and far-reaching biological and ecological questions. In addition, we stress that the use of semi-automated or full deep learning tools for animal identification is in its infancy and current results need to be evaluated in comparison with the logistical, financial, and potential ethical constraints of other commonly used sampling methods.

The specific goals for animal identification can vary greatly among studies and settings, objectives can generally be classified into two categories—applied and etiological—based on rationale, intention, and study design. Applied uses include those with the primary aims of describing, characterizing, and monitoring observed phenomena, including species distribution and abundance, animal movements and home ranges, or resource selection (Baird et al. 2008; Hughes and Burghardt 2017; Harris et al. 2020). These studies frequently adopt a top-down perspective in which the predominant focus is on groups (e.g., populations), with individuals simply viewed as units within the group and minimal interpretation of individual variability. As such, many of the modeling techniques employed for applied investigations, such as mark–recapture (Royle et al. 2013; Choo et al. 2020), are adept at incorporating quantified uncertainty in identification. However, reliable identification of individuals in applied studies is essential to accurate enumeration and differentiation when creating generalized models based on individual observations (Marin-Cudraz et al. 2019).

If not addressed and accounted for, misidentification can result in potential bias with substantial consequences for biological interpretations and conclusions (Rovero and Zimmermann 2016). For example, Johansson et al. (2020) demonstrated the potential ramifications of individual misclassification on capture–recapture-derived estimates of population abundance using camera trap photos of captive snow leopards. The authors employed a manual identification method wherein human observers were asked to identify individuals in images based on pelage patterns. Results indicated that observer misclassification resulted in population abundance estimates that were inflated by up to one-third. Hupman et al. (2018) also noted the potential for individual misidentification to result in under- or over-inflation of abundance estimates in a study exploring the use of photo-based mark–recapture for assessing population parameters of common dolphins. The authors found that inclusion of less distinctive individuals, for which identification was more difficult, resulted in seasonal abundance estimates that were substantially different (sometimes lower and sometimes higher) than when using photos of distinctive individuals only.

Many other questions, such as identifying the social hierarchy from passive observation, demand highly accurate identity tracking (Weissbrod et al. 2013; Schofield et al. 2019). Weissbrod et al. (2013) showed that due to the fine differences in social interactions even high identification rates of 99% can have measurable effects on results (as social hierarchy requires integration over long time scales). Though the current systems are not perfect, they can already outperform experts. For instance, Schofield et al. (2019) demonstrated (on a test set, for the frame-level identification task) that both novices (around 20%) and experts (around 42%) are outperformed by their system that reaches 84%, while only taking 60 ms versus 130 min and 55 min, for novices and experts, respectively.

These studies demonstrate the need to (1) be aware of the specific implications of potential errors in individual identification to their study conclusions and (2) choose an identification method that seeks to minimize misclassification to the extent practicable given their specific objectives and study design. While the techniques described in this review have already assisted in lowering identification error rates so as to mitigate this concern, for some applications they already reach sufficient accuracy (e.g., for conservation and management; Berger-Wolf et al. 2017; Crouse et al. 2017; Schofield et al. 2019; Guo et al. 2020), neuroscience and ethology (Romero-Ferrero et al. 2019; Lauer et al. 2021; Walter and Couzin 2021), and public engagement in zoos (Brookes and Burghardt 2020)). However, for many contexts, they have yet to reach the levels of precision associated with other applied techniques.

For comparison, genetic analyses are among the highest current standards for individual identification in applied investigations. While genotyping error rates caused by allelic dropouts, null alleles, false alleles, and so on. can vary between 0.2% and 15% per locus (Wang 2018); genetic analyses combine numerous loci to reach individual identification error rates of 1% (Weller et al. 2006; Baetscher et al. 2018). We stress that apart from accuracy many other variables should be considered, such as the relatively high logistical and financial costs associated with collecting and analyzing genetic samples, and the requirement to resample for re-identification. These results in sample sizes that are orders of magnitude smaller than many of the studies described above, with attendant decreases in explanatory/predictive power. Furthermore, repeated invasive sampling may directly or indirectly affect animal behavior. Minimally invasive sampling (MIS) techniques using feces, hair, feathers, remote skin biopsies, and so on offer the potential to conduct genetic identification in a less intrusive and less expensive manner (Carroll et al. 2018). MIS analyses are, however, vulnerable to genotyping errors associated with sample quality, with potential consequent ramifications to genotyping success rates (e.g., 87, 80, and 97% for Fluidigm SNP type assays of wolf feces, wildcat hair, and bear hair, respectively; Carroll et al. (2018) and references therein). These challenges, coupled with the increasing success rates and low financial and logistical costs of computer vision analyses, may effectively narrow the gap when selecting an identification technique. Furthermore, in some scenarios, the acceptable level of analytical error can be reduced without compromising the investigation of specific project goals, in which case biologists may find that current computer vision techniques are sufficiently robust to address applied biological questions in a manner that is low cost, logistically efficient, and can make use of pre-existing and archival images and video footage. In particular, the mark–recapture model, commonly employed in biological and ecological studies, lends itself well to a photo-identification adjustment (Royle et al. 2013; Choo et al. 2020). In a reworked format, the first photo would be a “capture,” the photo-identification would be the “mark,” and subsequent images would be the “recapture.” Other types of data or partial data, for example, time stamp or GPS location, may be incorporated to boost the success rate of photo-identification in mark–recapture models (Augustine et al. 2019, 2020).

Unlike their applied counterparts, etiological uses of individual identification do not seek to describe and characterize observed phenomena, but rather, to understand the mechanisms driving and influencing observed phenomena. This may include questions related to behavioral interactions, social hierarchies, mate choice, competition, altruism, and so on. (e.g., Parsons et al. 2009; Clapham et al. 2012; Weissbrod et al. 2013; Dell et al. 2014). Etiological studies are frequently based on a bottom-up perspective, in which the focus is on individuals, or the roles of individuals within groups, and interpretations of individual variability often play predominant roles (Díaz López 2020). As such, etiological investigations may seek to identify individuals in order to derive relationships among individuals, interpret outcomes of interactions between known individuals, assess and understand individuals’ roles in interactions or within groups, or characterize individual behavioral traits (Kelly et al. 1998; Constantine et al. 2007; Krasnova et al. 2014; Schofield et al. 2019). These studies are commonly done in laboratory settings, which present some study limitations. The ability to record data and assign it to an individual in the wild may be crucial to understand the origin and development of personality (Judy and Groothuis 2010; Dall et al. 2012). Characterizing behavioral variability of individuals is of great importance for understanding behavior (Roche et al. 2016). This has been highlighted in a meta-analysis that showed that a third of behavioral variation among individuals could be attributed to individual differences (Bell et al. 2009). The impact of repeatably measuring observations for single individuals can also be illustrated in the context of brain mapping. Repeated sampling of human individuals with fMRI is revealing fine-grained features of functional organization, which were previously unseen due to variability across the population (Braga and Buckner 2017). Overall, longitudinal monitoring of single individuals with powerful techniques such as omics (Chen et al. 2012) and brain imaging (Poldrack 2021) is heralding an exciting age for biology.

## Starting an animal identification project

For biological practitioners seeking to make sense of the possibilities offered by computer vision, the importance of inter-disciplinary collaborations with computer scientists cannot be overstated. Since the advent of high definition camera traps, some scientists find they have hours of opportunistically collected footage without a direct line of inquiry motivating the data collection. Collaboration with computer scientists can help to ensure the most productive analytical approach to using this footage to derive biological insights. Furthermore, by instituting collaborations early in the study design process, computer scientists can assist biologists in implementing image collection protocols that are specifically designed for use with deep learning analyses.

General considerations for starting an image-based animal identification project, such as which feature to focus on, are nicely reviewed by Kühl and Burghardt (2013). Although handcrafted features can be suited for certain species (e.g., zebras), deep learning has proven to be a more robust and general framework for image-based animal identification. However, at least a few thousand images with ideally multiple examples of each individual are needed, constituting the biggest limitation to obtaining good results. As such, data collection is a crucial part of the process. Discussion between biologists and computer scientists is fundamental and should be engaged before data collection. As previously mentioned, camera traps (Rovero and Zimmermann 2016; Caravaggi et al. 2017; Choo et al. 2020) can be used to collect data on a large spatial scale with little human involvement and less impact on animal behavior. Images from camera traps can be used both for model training and monitored for inference. The ability of camera traps to record multiple photos/videos of an individual allows multiple streams of data to be combined to enhance the identification process (as for localization [Beery et al. 2020]). Furthermore, camera traps minimize the potential influence of humans on animal behavior as seen in Schneider et al. (2019). However, noninvasive genetic sampling can be even less invasive, as camera traps can be heard and seen by animals (Meek et al. 2014).

Following image collection, researchers should employ tools to automatically sieve through the data to localize animals in pictures. Recent powerful detection models by Beery et al. (2019, 2020), trained on large-scale datasets of annotated images, are becoming available and generalize reasonably well to other datasets (Box 1). Those or other object detection models can be used out-of-the-box or finetuned to create bounding boxes around faces or bodies (Redmon et al. 2016; Liu et al. 2016; Ren et al. 2017), which can then be aligned by using pose estimation models (Mathis et al. 2020). Additionally, animal segmentation for background removal/identification can be beneficial.

Most methods require an annotated dataset, which means that one needs to label the identity of different animals on example frames; unsupervised methods are also possible (e.g., Turk and Pentland 1991; Crall et al. 2013; Otto et al. 2018). To start animal identification, a baseline model using triplet loss should be tried, which can be improved with different data augmentation schemes, combined with a classification loss, and/or expanded into more multi-task models. If attempting the classification approach, assigning classes to previously unseen individuals is not straightforward. Most works usually add a node for “unknown individual.” The evaluation pipeline to monitor the model’s performance has to be carefully designed to account for the way in which it will be used in practice. Of particular importance is how to split the dataset between training and testing subsets to avoid data leakage.

Ideally, one trains the model with the type of data that are used during deployment. In our experience generalization across different cameras is typically not ideal, which is why it is important to get results from different cameras during training if generalization is important. However, there are also computational methods to deal with this. For human reidentification, Zhong et al. (2018) used CycleGAN to transfer images from one camera style to another, although camera traps are perhaps too different. The generalization to other (similar) species is also a path to explore.

Other aspects to consider are the efficiency of models, even if identification is usually in an offline setting. Also, adding a “human-in-the-loop” approach, if the model does not perform perfectly, can still save time relative to a fully manual approach. For other considerations necessary to build a production ready system, readers are encouraged to look at Duyck et al. (2015), who created Sloop, with subsequent deep learning integration by Kshitij and Sai (2020) used for the identification of multiple species. Furthermore, Berger-Wolf et al. (2017) implemented different algorithms such as HotSpotter (Crall et al. 2013) in the Wild Me platform, which is actively used to identify a variety of species.

## Beyond image-based identification

As humans are highly visual creatures, it is intuitive that we gravitate to image-based identification techniques. Indeed, this preference may offer few drawbacks for applied uses of individual identification in which the researcher’s perspective is the primary lens through which discrimination and identification will occur. However, the interpretive objectives of etiological uses of identification add an additional layer of complexity that may not always favor a visually based method. When seeking to provide inference on the mechanisms shaping individual interactions, etiological applications must both (1) satisfy the researcher’s need to correctly identify known individuals and (2) attempt to interpret interactions based on an understanding of the sensory method by which the individuals in question identify and re-identify conspecifics (Tibbetts 2002; Thom and Hurst 2004; Tibbetts and Dale 2007).

Different species employ numerous mechanisms to engage in conspecific identification (e.g., olfactory, auditory, and chemosensory; Hagey and Macdonald 2003; Martin et al. 2008; Levréro et al. 2009). For example, previous studies have noted that giant pandas use olfaction for mate selection and assessment of competitors (Hagey and Macdonald 2003; Swaisgood et al. 2004). Conversely, Schneider et al. (2018) showed that Drosophila, which was previously assumed not to be strongly visually based, were able to engage in successful visual identification of conspecifics. Thus, etiological applications that seek to find mechanisms of animal identification must consider both the perspectives of the researcher and the individuals under study (much like Uexküll’s concept of *Umwelt* (Jakob 1992)), and researchers must embrace their roles as both observers and translators attempting to reconcile potential differences between human and animal perspectives.

Just as animals identify each other with different senses, future methods could also focus on other forms of data. Indeed, deep learning is not just revolutionizing computer vision, but problems as diverse as finding novel antibiotics (Stokes et al. 2020) and protein folding (Service 2020). Thus, we believe that deep learning will also strongly impact identification techniques for nonvisual data and make those techniques both logistically feasible and sufficiently noninvasive so as to limit disturbances to natural behaviors. Previous studies have employed techniques that are promising. For example, acoustic signals were used by Marin-Cudraz et al. (2019) for counting of rock ptarmigan, and by Dan et al. (2019) in an identification method that seems to generalize to multiple bird species. Furthermore, Kulahci et al. (2014) used deep learning to describe individual identification using olfactory–auditory matching in lemurs. However, this research was conducted on captive animals and further work is required to allow for application of these techniques in wild settings.

## Conclusions and outlook

Recent advances in computational techniques, such as deep-learning, have enhanced the proficiency of animal identification methods. Furthermore, end-to-end pipelines have been created, which allow for the reliable identification of specific individuals, with, in some cases, better than human-level performance. As most methods follow a supervised learning approach, the expansion of datasets is crucial for the development of new models, as is collaboration between computer science and biological teams in order to understand the applicable questions to both fields. Hopefully, this review has elucidated the fact that lines of inquiry to one group might have previously been unknown to the other, and that interdisciplinary collaboration offers a path for future methodological developments that are analytically nimble and powerful, but also applicable, dependable, and practicable to addressing real-world phenomena.

As we have illustrated, recent advances have contributed to the deployment of some methods, but many challenges remain. For instance, individual identification of unmarked, featureless animals such as brown bears or primates has not yet been achieved for hundreds of individuals in the wild. Likewise, discrimination of close siblings remains a challenging computer vision individual identification problem. How can the performance of animal individual identification methods be further improved?

Since considerably more attention and effort has been devoted to the computer vision question of human identification, versus animal identification, this vast literature can be used as a source of inspiration for improving animal individual identification techniques. Many human identification studies experiment with additional losses in a multi-task setting. For instance, whereas triplet loss maximizes inter-class distance, the center loss minimizes intra-class distance, and can be used in combination with the former to pull samples of the same class closer together (Wen et al. 2016). Furthermore, human identification studies demonstrate the use of spatio-temporal information to discard impossible matches (Wang et al. 2019). This idea could be used if an animal has just been identified somewhere and cannot possibly be at another distant location (using camera traps’ timestamps and GPS). Re-ranking the predictions has also been employed to improve performance in human-based studies using metric learning (Zhong et al. 2017). This approach aggregates the losses with an additional re-ranking based distance. Appropriate augmentation techniques can also boost performance (Zhong et al. 2020). In order to overcome occlusions, one can randomly erase rectangles of random pixels and random size from images in the training data set.

Applications involving human face recognition have also contributed significantly to the development of identification technologies. Human face datasets typically contain orders of magnitude more data (thousands of identities and many more images—e.g., the YouTube Faces dataset; Wolf et al. 2011) than those available for other animals. One of the first applications of deep learning to human face recognition was DeepFace, which used a classification approach (Yaniv et al. 2014). This was followed by Deep Face Recognition, which implemented a triplet loss bootstrapped from a classification network (Parkhi et al. 2015) and FaceNet by Schroff et al. (2015) which used triplet loss with semi hard mining on large batches. FaceNet achieved a top-1 score of 95.12% when applied to the YouTube Faces dataset. Some methods also showed promise for unlabeled datasets; Otto et al. (2018) proposed an unsupervised method to cluster *millions* of faces with approximate rank order metric. We note that this research also raises ethical concerns (Van Noorden 2020). Finally, benchmarks are important for advancing research and fortunately they are emerging for animal identification (Shuyuan et al. 2019), but more are needed.

Overall, broad areas for future efforts may include (1) improving the robustness of models to include other sensory modalities (consistent with conspecific identification inquiry) or movement patterns, (2) combining advanced image-based identification techniques with methods and technologies already commonly used in biological studies and surveys (e.g., remote sensing, population genetics, mark–recapture, etc.), and (3) creating larger benchmarks and datasets, for instance, via Citizen Science programs (e.g., eMammal; iNaturalist, Great Grevy’s Rally). While these areas offer strong potential to foster analytical and computational advances, we caution that future advancements should not be dominated by technical innovation, but rather, technical development should proceed in parallel with, or be driven by, the application of novel and meaningful biological questions. Following a question-based approach will assist in ensuring the applicability and utility of new technologies to biological investigations and potentially mitigate against the use of identification techniques in suboptimal settings.

## Funding

Support for M.V., B.R., N.W., and B.P.H. was provided by Alaska Education Tax Credit funds contributed by the At-Sea Processors Association and the Groundfish Forum.

Box 1 Other relevant computer vision tasks

Deep learning has greatly advanced many computer vision tasks relevant to biology (LeCun et al. 2015; Norouzzadeh et al. 2018; Schneider et al. 2019; Mathis et al. 2020; Wu et al., 2020). For example:

**Animal detection**: A subset of object detection, the branch of computer vision that deals with the tasks of localizing and classifying objects in images or videos. Current state-of-the-art methods for object recognition usually employ anchor boxes, which represent the target location, size, and object class, such as in EfficientDet (Tan et al. 2020), or newly end-to-end like, as in DETR (Carion et al. 2020). Of particular interest for camera-trap data is the powerful MegaDetector (Beery et al. 2019), which is trained on more than 1 million labeled animal images and also actively updated.^2^ Also relevant for camera-traps, Beery et al. (2020) developed attention-based detectors that can reason over multiple frames, integrating contextual information and thereby strongly improving performance. Various detectors have been used in the animal identification pipeline (Redmon et al. 2016; Liu et al. 2016; Ren et al. 2017), which, however, are no longer state-of-the-art on detection benchmarks.

**Animal species classification**: The problem of classifying *species* based on pictures (Villa et al. 2017; Norouzzadeh et al. 2018). As performance is correlated to the amount of training data, most recently synthetic animals have been used to improve the classification of rare species, which is a major challenge (Beery et al. 2020).

**Pose estimation**: The problem of estimating the pose of an entity from images or videos. Algorithms can be top down, where the individuals are first localized, as in Wang et al. (2020) or bottom up (without prior localization) as in Cheng et al. (2020). Recently, several user-friendly and powerful software packages for pose estimation with deep learning for animals were developed, reviewed in Mathis et al. (2020); real-time methods for closed-loop feedback are also available (Kane et al. 2020).

**Alignment**: In order to effectively compare similar regions and orientations—animals (in pictures) are often aligned using pose estimation or object recognition techniques.

Box 2 Deep Learning terms glossary

**Machine and deep learning**: Machine learning seeks to develop algorithms that automatically detect patterns in data. These algorithms can then be used to uncover patterns, to predict future data, or to perform other kinds of decision making under uncertainty (Murphy 2012). Deep learning is a subset of machine learning that utilizes artificial neural networks with multiple layers as part of the algorithms. For computer vision problems, ConvNets are the de-facto standard building blocks. They consist of stacked convolutional filters with learnable weights (i.e., connections between computational elements). Convolutions bake translation invariance into the architecture and decrease the number of parameters due to weight sharing, as opposed to ordinary fully-connected neural networks (Krizhevsky et al. 2012; LeCun et al. 2015; He et al. 2016). SVMs: A powerful classification technique, which learns a hyperplane to separate data points in feature spaces; nonlinear SVMs also exist (Murphy 2021). Principal component analysis (PCA): An unsupervised technique that identifies a lower dimensional linear space, such that the variance of the projected data is maximized (Murphy 2021); Turk and Pentland (1991) used it for face recognition.

**Classification network**: A neural network that directly predicts the class of an object from inputs (e.g., images). The outputs have a confidence score as to whether they correspond to the target. Often trained with a cross entropy loss, or other prediction error based losses (Krizhevsky et al. 2012; Chatfield et al. 2014; He et al. 2016).

**Metric learning**: A branch of machine learning which consists in learning how to measure similarity and distance between data points (Bellet et al. 2013)—common examples include siamese networks and triplet loss.

**Siamese networks**: Two identical networks that consider a pair of inputs and classify them as similar or different, based on the distance between their embeddings. It is often trained with a contrastive loss, a distance-based loss, which pulls positive (similar) pairs together and pushes negative (different) pairs away:

$$\ell \left(W,Y,{\vec{X}}_{1},{\vec{X}}_{2}\right)=(1-Y)\frac{1}{2}{\left(D_{W}\right)}^{2}+(Y)\frac{1}{2}{\left\{\max\left(0,m-D_{W}\right)\right\}}^{2}$$

where *D_W_* is any metric function parametrized by *W*, *Y* is a binary variable that represents if $\left({\vec{X}}_{1},{\vec{X}}_{2}\right)$ is a similar or dissimilar pair (Hadsell et al. 2006).

**Triplet loss**: As opposed to pairs in siamese networks, this loss uses triplets; it tries to bring the embedding of the anchor image closer to another image of the same class than to an image of a different class by a certain margin. In its naive form

$$\ell =\max(d_{a,p}-d_{a,n}+\text{margin},0)$$

where $d_{a,p}$ ($d_{a,n}$) is the distance from the anchor image to its positive (negative) counterpart. As shown in Hermans et al. (Hermans et al. 2017), models with this loss are difficult to train, and triplet mining (heuristics for the most useful triplets) is often used. One solution is semi-hard mining, e.g., showing moderately difficult samples in large batches, as in Schroff et al. (2015). Another more efficient solution is the batch hard variant introduced in (Hermans et al. 2017), where one samples multiple images for a few classes, and then keeps the hardest (i.e., furthest in the feature space) positive and the hardest negative for each class to compute the loss. Mining the easy positives (very similar pairs; Hong et al. 2020), has recently proven to obtain good results.

**mAP**: With precision defined as $\frac{\text{TP}}{\text{TP}+\text{FP}}$ (TP: true positives, FP: false positives), and recall defined as $\frac{\text{TP}}{\text{TP}+\text{FN}}$ (FN: false negative), the average precision is the area under the precision recall curve (see Murphy (2021) for more information), and the mAP is the mean for all queries.

**Transfer learning**: The process when models are initialized with features, trained on a (related) large-scale annotated dataset, and then finetuned on the target task. This is particularly advantageous when the target dataset consists of only few labeled examples (Mathis et al. 2020; Zhuang et al. 2020). ImageNet is a large-scale object recognition data set (Russakovsky et al. 2015) that was particularly influential for transfer learning. As we outline in the main text, many methods use ConvNets pre-trained on ImageNet such as AlexNet (Krizhevsky et al. 2012), VGG (Chatfield et al. 2014), and ResNet (He et al. 2016).

## Footnotes

1 In publications, the terminology *re-identification* is often used interchangeably. In this review we posit that *re-identification* refers to the recognition of (previously) known individuals, hence we use *identification* as the more general term.

2
https://github.com/microsoft/CameraTraps/blob/master/megadetector.md

3 For the purposes of this review, we forgo discussion of individual identification in the context of the agricultural sciences, as circumstances differ greatly in those environments. However, we note that there is an emerging body of computer vision for the identification of livestock (Qiao et al. 2020; William et al. 2021).

## Notes

From the symposium “Spatiotemporal dynamics of animal communication” presented at the virtual annual meeting of the Society for Integrative and Comparative Biology, January 3–7, 2021.
